# Aging Reduces Estradiol Protection Against Neural but Not Metabolic Effects of Obesity in Female 3xTg-AD Mice

**DOI:** 10.3389/fnagi.2020.00113

**Published:** 2020-05-05

**Authors:** Amy Christensen, Jiahui Liu, Christian J. Pike

**Affiliations:** Davis School of Gerontology, University of Southern California, Los Angeles, CA, United States

**Keywords:** aging, Alzheimer’s disease, β-amyloid, estrogen, hormone therapy, microglia, obesity

## Abstract

Vulnerability to Alzheimer’s disease (AD) is increased by several risk factors, including midlife obesity, female sex, and the depletion of estrogens in women as a consequence of menopause. Conversely, estrogen-based hormone therapies have been linked with protection from age-related increases in adiposity and dementia risk, although treatment efficacy appears to be affected by the age of initiation. Potential interactions between obesity, AD, aging, and estrogen treatment are likely to have significant impact on optimizing the use of hormone therapies in postmenopausal women. In the current study, we compared how treatment with the primary estrogen, 17β-estradiol (E2), affects levels of AD-like neuropathology, behavioral impairment, and other neural and systemic effects of preexisting diet-induced obesity in female 3xTg-AD mice. Importantly, experiments were conducted at chronological ages associated with both the early and late stages of reproductive senescence. We observed that E2 treatment was generally associated with significantly improved metabolic outcomes, including reductions in body weight, adiposity, and leptin, across both age groups. Conversely, neural benefits of E2 in obese mice, including decreased β-amyloid burden, improved behavioral performance, and reduced microglial activation, were observed only in the early aging group. These results are consistent with the perspective that neural benefits of estrogen-based therapies require initiation of treatment during early rather than later phases of reproductive aging. Further, the discordance between E2 protection against systemic versus neural effects of obesity across age groups suggests that pathways other than general metabolic function, perhaps including reduced microglial activation, contribute to the mechanism(s) of the observed E2 actions. These findings reinforce the potential systemic and neural benefits of estrogen therapies against obesity, while also highlighting the critical role of aging as a mediator of estrogens’ protective actions.

## Introduction

Alzheimer’s disease (AD) is a progressive neurodegenerative disorder that is the most common cause of dementia. While aging is the strongest risk factor for the disease, the development of AD is affected by numerous modifiable and non-modifiable risk factors (Hersi et al., 2017). One important and highly prevalent modifiable AD risk factor is obesity; people who are overweight or obese in midlife are at significantly increased risk for development of AD and related dementias in late life (Alford et al., 2018). Obesity-related conditions, including metabolic syndrome and type 2 diabetes, are also independently associated with increased dementia risk (Misiak et al., 2012; Bangen et al., 2019). Because there are no effective disease-modifying therapeutics for AD, a promising interventional approach is to reduce vulnerability to AD by mitigating the effects of modifiable risk factors including obesity.

Another important AD risk factor is female sex. Women have a higher lifetime risk of AD than men as well as greater prevalence, with women accounting for approximately two-thirds of AD cases (Mazure and Swendsen, 2016; Alzheimer’s Association, 2018). There are numerous sex differences in the development, progression and manifestation of AD that likely contribute to the disease’s female bias (Pike, 2017). One significant component is the depletion of sex steroid hormones, in particular 17β-estradiol (E2), which occurs as a consequence of menopause. Estradiol exerts numerous beneficial effects in tissues throughout the body, including the brain, such that the loss of estradiol can increase risks for dysfunction and disease (Clegg et al., 2017; Merlo et al., 2017). In transgenic mouse models of AD, depletion of estrogens by ovariectomy accelerates the onset and progression of AD-like neuropathology and cognitive impairment, whereas treatment with E2 protects against these effects (Pike et al., 2009; Li and Singh, 2014). In women, early surgical menopause and other causes of reduced lifetime exposure to estrogens are associated with elevated AD risk (Bove et al., 2014; Pike, 2017; Jang et al., 2018). Whether estrogen-based hormone therapies (HT) significantly reduce AD risk in postmenopausal women is uncertain, with conflicting findings across studies (Yaffe et al., 1998; Hamoda et al., 2016; Lobo et al., 2016; Lobo, 2017). One hypothesis that appears to explain inconsistencies in the literature is that HT must be administered near the time of menopause in order to protect against dementia (Depypere et al., 2016; Lobo, 2017; Marjoribanks et al., 2018). Thus, reproductive and/or chronological age may be critical variables in the efficacy of HT as preventive strategy for AD.

Importantly, obesity, female sex, and reproductive and chronological aging are interactive AD risk factors. For example, estrogen depletion resulting from menopause is linked not only with heightened AD risk, but also increased adiposity and risk for metabolic dysfunction, which in turn are associated with elevated AD risk (Christensen and Pike, 2015). Estrogens can reduce adiposity and improve both metabolic symptoms (Brown et al., 2010) and neural functions (Frick et al., 2015) but are also predicted to exhibit reduced efficacy with increasing age (Morrison et al., 2006). In a recent investigation of these interactions in female 3xTg-AD mice, we found that E2-based HT initiated at the onset of diet-induced obesity reduced AD-related pathology and cognitive impairment in early but not late middle age (Christensen and Pike, 2017). These findings are consistent with the conclusion that HT protects against the development of obesity-related neural impairment in AD mice, with the preventive outcomes exhibiting age-dependence. What remains unclear is whether estrogens can also protect against pre-existing obesity and thus function as a treatment for a modifiable risk factor of AD. The current study examines this latter question. Female 3xTg-AD mice were maintained on an obesogenic diet for 2 months beginning in early middle age (early-MA) prior to the onset of reproductive aging or in late middle-age (late-MA) well beyond the cessation of ovarian cycling, then treated with estradiol for 2 months in the continued presence of obesogenic diet. The data demonstrate that estradiol reduces metabolic consequences of obesity across age groups, but significantly improves aspects of AD-related pathology and cognitive impairment only when administered in early middle age.

## Materials and Methods

### Animals

**FIGURE 1:**
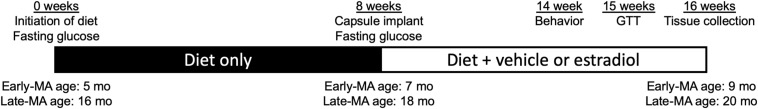
Schematic of experimental time course. The 16-week experimental period included an initial 8-week stage (filled bar) in which mice in early (Early-MA) and late middle age (Late-MA) were randomized to groups maintained on either Control or HFD. After week 8, the second 8-week stage began (open bar) with animals receiving either a vehicle- or estradiol-filled Silastic capsule that was retained through the end of the experiment. The timing of select outcomes is also indicated. At Week 14, animals were tested for spontaneous alternation behavior. At Week 15, a glucose tolerance test (GTT) was administered to assess metabolic function. At Week 16, animals were euthanized and tissues collected. Ages of Early-MA and Late-MA groups at each stage are indicated along the bottom of the diagram.

A colony of 3xTg-AD mice (Oddo et al., 2003) were maintained at the University of Southern California with *ad libitum* access to chow and water under a 12 h light/dark schedule (lights on at 6:00 AM). Female 3xTg-AD mice were randomized to experimental groups that were maintained on either control (10% calories from fat and 7% from sugar; catalog #D12450Ji, Research Diets, Inc., New Brunswick, NJ, United States) or high-fat diet (60% calories from fat and 7% from sugar; catalog #D12492i, Research Diets, Inc) for 16-weeks. Experimental groups were exposed to diet between ages 5–9 months (*n* = 8/group) or 16–20 (*n* = 6–7/group) months, chronological age spans in female mice that correspond to early stages of both middle age (early-MA) and reproductive senescence versus late stages of middle age (late-MA) and reproductive senescence, respectively (Nelson et al., 1982; Felicio et al., 1984; Finch, 2014). Animals were weighed weekly during the diet exposure period. After the first 8 weeks of diet, animals were anesthetized with the inhalant isoflurane (3%), then implanted subcutaneously (between the shoulder blades) with a Silastic capsule (1.47 mm ID x 1.96 mm OD; Dow Corning, Midland, MI, United States). Each capsule had a total length of 7 mm with the inner 3 mm packed with cholesterol (vehicle) or a 1:3 mixture of E2 to cholesterol. In 5 months-old, gonadally intact female mice maintained on control diet, E2 capsules yielded a statistically non-significant trend toward increased plasma E2 (114.5 ± 40.9 pg/mL) relative to vehicle-treated (42.1 ± 32.7 pg/mL) mice (*n* = 4–5, *t*-test: *p* = 0.23). Eight weeks following capsule implantation (16 weeks after initiation of diet), all animals were euthanized. The experimental design is summarized in Figure 1. Brains were immersion fixed in 4% paraformaldehyde for 72 h and subsequently stored at 4°C. Plasma was collected and stored in aliquots at −80°C. Visceral and retroperitoneal fat pads were dissected, weighed, and stored at −80°C. All procedures were conducted in accordance with the guidelines set forth by the university’s Institutional Animal Care and Use Committee and under the supervision of university veterinarians.

### Glucose Measurements

Fasting glucose (16 h overnight fast) was measured at weeks 0, 8, and 15 of the experimental period (Figure 1). Five microliters of blood were collected on a glucose test strip and assayed using a Precision Xtra glucose monitor (Abbott Laboratories). A glucose tolerance test was performed after 15 weeks of diet (7 weeks after the start of E2 or vehicle treatment). In brief, animals were orally gavaged with 2 g/kg D-glucose and blood glucose levels were measured at 0, 15, 30, 60, and 120 min thereafter.

### Spontaneous Alternation Behavior

At week 14, animals were behaviorally assessed using the spontaneous alternation behavior test in a Y-maze (Figure 1). The spontaneous alternation test is dependent upon the hippocampus and other limbic structures (Lalonde, 2002) and assesses spatial memory and attention toward novelty (Hughes, 2004). Animals were allowed to acclimate to the behavior room for 30 min prior to testing. Next, animals were placed in the long arm of a Y-maze facing away from the other arms to start the test. Arm entries (at least two paws placed into an arm) were recorded for 5 min. Animals with fewer than 10 arm entries were excluded from analysis. Percent spontaneous alternation was calculated as number of correct triplicates divided by total number of triplicate arm entries.

### Immunohistochemistry

Fixed hemibrains were sectioned exhaustively in the horizontal plane at 40 μm using a vibratome (Leica Biosystems). Sections were stored singly in PBS with 0.03% sodium azide at 4°C until immunohistochemistry was performed. Every eighth section (from a total of ∼100 per brain) was immunostained for β-amyloid (Aβ) as previously described (Christensen and Pike, 2017). In brief, tissue sections containing hippocampus were pretreated with 95% formic acid for 5 min, then washed three times for 5 min in TBS, followed by a 10 min rinse with an endogenous peroxidase blocking solution. Next, sections were rinsed in TBS/0.1% Triton-X before being incubated for 30 min in a blocking solution consisting of TBS/2% bovine serum albumin. Sections were incubated overnight at 4°C with anti-Aβ antibody (1:300; Life Technologies; Cat #71-5800) diluted in blocking solution. Immunostaining was also conducted in the absence of formic acid pretreatment with the following primary antibodies: doublecortin (1:2500; Santa Cruz) as a marker of new neurons, clone AT8 (1:750; Thermo) for phosphorylated tau (phospho-tau), Iba-1 (1:2000; Wako) for microglia. Sections incubated in primary antibody were washed and then incubated with the appropriate secondary antibody (Vector Laboratories) for 1 h and processed for diaminobenzidene visualization using Vectastain ABC Elite kit (Vector Laboratories). Stained sections were air-dried overnight, dehydrated in a series of graded alcohols, then coverslipped with Krystalon (EMD Millipore).

### β-Amyloid Load

To assess Aβ load, the percentage area of Aβ immunoreactivity was determined, as previously described (Christensen and Pike, 2018). In brief, non-overlapping high magnification images were collected from the subiculum (three fields/section) and CA1 hippocampal subfield (three fields/section) across four tissue sections per brain, for a total of ∼24 images per brain. Images were digitally captured using an Olympus BX50 microscope and DP74 camera paired with a computer running CellSens software (Olympus). Images were converted to grayscale and thresholded using NIH ImageJ 1.50i to yield binary images separating positive and negative immunostaining. Aβ load was calculated as the percentage of the total pixels that was positively immunolabeled.

### Quantification of Immunolabeled Cells

Doublecortin- and tau-immunoreactive cells were counted from 4 sections per brain. Positive labeling was defined as cells that were darkly stained across the majority of the soma. Doublecortin-labeled cells were counted across the entire dentate gyrus. Tau-positive cells were counted across the entire subiculum and CA1 regions of the hippocampus. Microglial activation was based on morphological analysis of Iba-1 immunoreactive cells, as previously described (Christensen and Pike, 2017, 2018). Density of Iba-1 immunoreactive cells in the hippocampus was estimated by two-dimensional counts. Briefly, an Olympus BX50 microscope equipped with a motorized stage and computer-guided CASTGrid software (Olympus) was used for unbiased sampling. In four sections per animal, the area containing the subiculum and CA1–CA3 subregions of the hippocampus (excluding the dentate gyrus) was sampled at high magnification. Within each field, cells within a counting frame (3000 μm^2^) were used for analysis. Microglia were classified as either type 1, (many thin, ramified processes), type 2 (short, thick processes and a rod-shaped cell body), or type 3 (no or few short non-ramified processes or many filapodial processes) cells. Type 2 and 3 cells were considered to exhibit an activated microglia morphological phenotype.

### ELISAs

Estradiol concentration in plasma was measured using an estradiol ELISA (Calbiotech) according to the manufacturer’s instructions. Plasma leptin was measured using a leptin ELISA (Millipore), according to the manufacturer’s protocol.

### PCR

For RNA extractions, visceral fat pads were dissociated using a dounce homogenizer in TRIzol reagent (Invitrogen), following the manufacturer’s protocol except that a 10 min 12000 × *g* spin was added prior to the addition of chloroform to reduce lipid content. The resultant RNA pellet was treated with RNase-free DNase I (Epicentre) for 30 min at 37°C, and a phenol/chloroform extraction was performed to isolate RNA. The iScript cDNA synthesis system (Bio-Rad) was used to reverse transcribe cDNA from 1 μg of purified RNA. Real-time quantitative PCR was performed on the resulting cDNA using ssoAdvanced Universal SYBR Green Supermix (Bio-Rad) and a Bio-Rad CFX96 Touch Real-Time PCR Detection System. As a general rule, samples from all experimental groups were run on each plate and ΔΔCt results were compared to relative to the early middle age control mice (control diet + vehicle capsule). PCR was performed using the following primer pairs: CD68 (forward: TTCTGCTGTGGAAATGCAAG and reverse: AGAGG GGCTGGTAGGTTGAT) F4/80 (forward: TGCATCTAGCAA TGGACAGC and reverse: GCCTTCTGGATCCATTTGAA), interleukin-6 (IL-6; forward: AGTTGCCTTCTTGGGACTGA and reverse: TCCACGATTTCCCAGAGAAC), succinate dehydrogenase complex, subunit A (SDHA; forward: ACACA GACCTGGTGGAGACC and reverse: GGATGGGCTTGGAGT AATCA) and hypoxanthine guanine phosphoribosyltransferase (HPRT; forward: AAGCTTGCTGGTGAAAAGGA and reverse: TTGCGCTCATCTTAGGCTTT). Expression levels of CD68, F4/80, and IL-6 were normalized to the average expression of the two reference markers, SDHA and HPRT.

### Statistics

All data are reported as the mean ± the standard error of the mean. Data were analyzed using GraphPad Prism version 8. Most data were statistically analyzed using two-way ANOVA followed by Tukey’s *post hoc* tests when appropriate. Three-way repeated measure ANOVA followed by Tukey’s *post hoc* tests was used to analyze data measured over time (body weight, fasting glucose, GTT) and to compare the effects of age, diet and hormone treatment. Statistical analyses are listed in Tables 1, 2.

**TABLE 1:** Statistical analyses of data within age groups.

**Figure**	**Main Effects, Interactions**	***Post hoc* comparisons**
2A	*F*_time_(1.80,50.3) = 76.3, *p* < 0.0001 *F*_diet_(1,28) = 41.4, *p* < 0.0001 *F*_time × diet_(4,112) = 41.4, *p* < 0.0001 *F*_time × hormone_(4,112) = 8.11, *p* < 0.0001 *F*_time × diet × hormone_(4,112) = 9.25, *p* < 0.0001	8 weeks: Con + Veh vs. HFD + Veh, *p* = 0.08 Con + E2 vs. HFD + E2, *p* = 0.08 12 weeks: Con + Veh vs. HFD + Veh, *p* < 0.05 Con + E2 vs. HFD + E2, *p* = 0.23 16 weeks: Con + Veh vs. HFD + Veh, *p* < 0.01 Con + E2 vs. HFD + E2, *p* = 0.20
2B	*F*_diet_(1,28) = 54.5, *p* < 0.0001	Con + Veh vs. HFD + Veh, *p* < 0.0001 Con + E2 vs. HFD + E2, *p* < 0.0001
2C	*F*_hormone_(1,28) = 24.7, *p* < 0.0001 *F*_interaction_(1,28) = 25.7, *p* < 0.0001	Con + Veh vs. HFD + Veh, *p* = 0.001 Con + E2 vs. HFD + E2, *p* < 0.05 HFD + Veh vs. HFD + E2, *p* < 0.0001
2D	*F*_diet_(1,28) = 61.5, *p* < 0.0001 *F*_hormone_(1,28) = 20.7, *p* < 0.0001 *F*_interaction_(1,28) = 10.7, *p* < 0.01	Con + Veh vs. HFD + Veh, *p* < 0.0001 Con + E2 vs. HFD + E2, *p* < 0.05 HFD + Veh vs. HFD + E2, *p* < 0.0001
2E	*F*_diet_(1,28) = 62.5, *p* < 0.0001 *F*_hormone_(1,28) = 29.8, *p* < 0.0001 *F*_interaction_(1,28) = 21.1, *p* < 0.0001	Con + Veh vs. HFD + Veh, *p* < 0.0001 Con + E2 vs. HFD + E2, *p* < 0.0001 HFD + Veh vs. HFD + E2, *p* < 0.0001
2F	*F*_time_(2.49,57.3) = 6.53, *p* < 0.01 *F*_diet_(1,23) = 35.1, *p* < 0.0001 *F*_hormone_(1,23) = 4.13, *p* = 0.054 *F*_time × diet_(4,92) = 23.8, *p* < 0.0001 *F*_time × hormone_(4,92) = 9.7, *p* < 0.0001 *F*_time × diet × hormone_(4,92) = 10.6, *p* < 0.0001	8 weeks: Con + Veh vs. HFD + Veh, *p* < 0.05 Con + E2 vs. HFD + E2, *p* = 0.07 12 weeks: Con + Veh vs. HFD + Veh, *p* = 0.056 Con + E2 vs. HFD + E2, *p* = 0.22 16 weeks: Con + Veh vs. HFD + Veh, *p* = 0.06 Con + E2 vs. HFD + E2, *p* = 0.07
2G	*F*_diet_(1,23) = 52.5, *p* < 0.0001	Con + Veh vs. HFD + Veh, *p* < 0.0001 Con + E2 vs. HFD + E2, *p* < 0.001
2H	*F*_diet_(1,23) = 5.5, *p* < 0.05 *F*_hormone_(1,23) = 5.4, *p* < 0.05 *F*_interaction_(1,23) = 9.3, *p* < 0.001	Con + E2 vs. HFD + E2, *p* < 0.01 HFD + Veh vs. HFD + E2, *p* < 0.01
2J	*F*_diet_(1,23) = 13.2, *p* < 0.01 *F*_hormone_ (1,23) = 20.9, *p* < 0.0001 *F*_interaction_(1,23) = 5.9, *p* < 0.05	Con + Veh vs. HFD + Veh, *p* < 0.01 HFD + Veh vs. HFD + E2, *p* < 0.001
2I	*F*_diet_(1,23) = 15.1, *p* < 0.001 *F*_hormone_(1,23) = 18.9, *p* < 0.001 *F*_interaction_(1,23) = 6.4, *p* < 0.05	Con + Veh vs. HFD + Veh, *p* < 0.001 HFD + Veh vs. HFD + E2, *p* < 0.001
3A	*F*_time_(1.86,52.1) = 11.6, *p* < 0.0001 *F*_diet_(1,28) = 3.74, *p* = 0.06 *F*_time × diet_(2,56) = 5.11, *p* < 0.01	No significant differences
3B	*F*_time_(2.73,76.3) = 137, *p* < 0.0001 *F*_diet_(1,28) = 6.53, *p* < 0.05 *F*_time × diet_(4,112) = 4.13, *p* < 0.01	No significant differences
3C	*F*_diet_(1,28) = 25.3, *p* < 0.0001 *F*_hormone_(1,28) = 11.0, *p* < 0.01 *F*_interaction_(1,28) = 8.1, *p* < 0.01	Con + Veh vs. HFD + Veh, *p* < 0.0001 HFD + Veh vs. HFD + E2, *p* < 0.001
3D	*F*_diet_(1,28) = 8.0, *p* < 0.01	No significant differences
3E	*F*_time_(1.98,45.6) = 8.6, *p* < 0.001 *F*_diet_(1,23) = 6.5, *p* < 0.05	No significant differences
3F	*F*_time_(2.85,57.0) = 194, *p* < 0.0001 *F*_time × diet_(4,80) = 4.38, *p* < 0.01 *F*_diet × hormone_(1,20) = 11.0, *p* < 0.01 *F*_time × diet × hormone_(4,80) = 3.15, *p* < 0.05	60 min: Con + Veh vs. HFD + Veh, *p* < 0.001
3G	*F*_diet_(1,23) = 9.5, *p* < 0.01 *F*_hormone_(1,23) = 14.0, *p* < 0.01 *F*_interaction_(1,23) = 6.8, *p* < 0.05	Con + Veh vs. HFD + Veh, *p* < 0.01 HFD + Veh vs. HFD + E2, *p* < 0.001
3H	*F*_interaction_(1,20) = 16.1, *p* < 0.001	Con + Veh vs. HFD + Veh, *p* < 0.05 Con + Veh vs. Con + E2, *p* < 0.05
4A	*F*_hormone_(1,22) = 12.9, *p* < 0.01	HFD + Veh vs. HFD + E2, *p* < 0.05
4B	No significant main effects	
4C	No significant main effects	
4D	No significant main effects	
5E	*F*_interaction_(1,28) = 9.9, *p* < 0.01	HFD + Veh vs. HFD + E2, *p* < 0.05
5F	*F*_hormone_(1,28) = 3.1, *p* = 0.09 [without outlier: *F*_hormone_(1,27) = 3.4, *p* = 0.07]	
5K	No significant main effects	
5L	*F*_diet_(1,23) = 6.3, *p* < 0.05	Con + E2 vs. HFD + E2, *p* < 0.05
6A	No significant main effects	
6B	*F*_hormone_(1,28) = 16.6, *p* < 0.001 *F*_interaction_(1,28) = 4.2, *p* < 0.05	HFD + Veh vs. HFD + E2, *p* < 0.001
6C	*F*_diet_(1,22) = 4.8, *p* < 0.05	No significant differences
6D	*F*_diet_(1,22) = 6.0, *p* < 0.05	No significant differences
7A	*F*_diet_(1,28) = 7.1, *p* < 0.05	Con + Veh vs. HFD + Veh, *p* < 0.05
7B	*F*_diet_(1,28) = 7.5, *p* < 0.05	Con + Veh vs. HFD + Veh, *p* < 0.05
7C	*F*_diet_(1,28) = 4.6, *p* < 0.05	No significant differences
7D	*F*_diet_(1,23) = 9.8, *p* < 0.01	Con + Veh vs. HFD + Veh, *p* < 0.05
7E	*F*_diet_(1,23) = 4.4, *p* < 0.05	No significant differences
7F	*F*_hormone_(1,23) = 5.2, *p* < 0.05	No significant differences

**TABLE 2:** Statistical analyses of data between age groups.

**Figure**	**Main effects, Interactions**	***Post hoc* comparisons**
2A and 2F	*F*_time × age × diet_(4,104) = 55.8, *p* < 0.0001 *F*_time × age × hormone_(4,104) = 55.7, *p* < 0.0001	0 weeks: E-MA Con + Veh vs. L-MA Con + Veh, *p* < 0.001 E-MA HFD + Veh vs. L-MA HFD + Veh, *p* < 0.001 E-MA HFD + E2 vs. L-MA HFD + E2, *p* < 0.05 4 weeks: E-MA HFD + Veh vs. L-MA HFD + Veh, *p* < 0.0001 E-MA HFD + E2 vs. L-MA HFD + E2, *p* < 0.01 8 weeks: E-MA HFD + Veh vs. L-MA HFD + Veh, *p* < 0.0001 E-MA HFD + E2 vs. L-MA HFD + E2, *p* < 0.05 12 weeks: E-MA HFD + Veh vs. L-MA HFD + Veh, *p* < 0.01 16 weeks: E-MA HFD + Veh vs. L-MA HFD + Veh, *p* < 0.05
2B and 2G	*F*_age × diet_(1,26) = 4.4, *p* < 0.05	No significant differences
2C and 2H	*F*_age × diet_(1,26) = 24.6, *p* < 0.0001 *F*_age × hormone_(1,27) = 29.7, *p* < 0.0001	E-MA HFD + Veh vs. L-MA HFD + Veh, *p* < 0.001 E-MA HFD + E2 vs. L-MA HFD + E2, *p* < 0.05
2D and 2I	No significant main effects	
2E and 2J	No significant main effects	
3A and 3E	*F*_time × age × diet_(2,52) = 9.5, *p* < 0.001 *F*_time × age × hormone_(2,52) = 18.6, *p* < 0.0001	No significant differences
3B and 3F	*F*_time × age × diet_(4,96) = 174.5, *p* < 0.0001 *F*_time × age × hormone_(4,96) = 151.0, *p* < 0.0001	No significant differences
3C and 3G	No significant main effects	
3D and 3H	*F*_age × diet_(1,24) = 3.5, *p* = 0.08	No significant differences
4A and 4C	No significant main effects	
4B and 4D	*F*_age × diet_(1,26) = 28.5, *p* < 0.0001	E-MA Control + Veh vs. L-MA Control + Veh, *p* < 0.001 E-MA HFD + Veh vs. L-MA HFD + Veh, *p* < 0.05
5E and 5K	*F*_age × diet_(1,26) = 57.2, *p* < 0.0001	E-MA Control + Veh vs. L-MA Control + Veh, *p* < 0.001 E-MA HFD + Veh vs. L-MA HFD + Veh, *p* < 0.0001
5F and 5L	*F*_age × diet_(1,26) = 94.4, *p* < 0.0001	E-MA Control + Veh vs. L-MA Control + Veh, *p* < 0.0001 E-MA HFD + Veh vs. L-MA HFD + Veh, *p* < 0.0001
6A and 6C	*F*_age × diet_(1,25) = 28.0, *p* < 0.0001 *F*_age × hormone_(1,26) = 69.6, *p* < 0.0001	E-MA Control + Veh vs. L-MA Control + Veh, *p* < 0.05 E-MA HFD + Veh vs. L-MA HFD + Veh, *p* < 0.01 E-MA HFD + E2 vs. L-MA HFD + E2, *p* < 0.0001
6B and 6D	*F*_age × diet_(1,25) = 44.6, *p* < 0.0001 *F*_age × hormone_(1,26) = 141.3, *p* < 0.0001	E-MA Control + Veh vs. L-MA Control + Veh, *p* < 0.0001 E-MA HFD + Veh vs. L-MA HFD + Veh, *p* < 0.01 E-MA HFD + E2 vs. L-MA HFD + E2, *p* < 0.0001
7A and 7D	*F*_diet_(1,51) = 17.3, *p* < 0.0001 *F*_diet × hormone_(1,51) = 5.4, *p* < 0.05	No significant differences
7B and 7E	*F*_diet_(1,51) = 11.1, *p* < 0.01 *F*_diet × hormone_(1,51) = 4.4, *p* < 0.05	No significant differences
7C and 7F	*F*_diet_(1,51) = 3.1, *p* = 0.09 *F*_age × diet_(1,51) = 3.3, *p* = 0.08	No significant differences

*E-MA, early middle age; L-MA, late middle age.*

## Results

### Estradiol Affects Body Mass and Adiposity in Both Early and Late Middle Age

We sought to determine how the protective efficacy of E2 is affected by preexisting obesity across the transitions associated with middle age, including reproductive senescence. To accomplish this, female 3xTg-AD mice were implanted with a Silastic capsule containing either vehicle (Veh) or E2 halfway through a 16-week exposure to either a control (Con) or high-fat (HFD) diet during ages corresponding to early (early-MA) or late (late-MA) middle age (Figure 1).

**FIGURE 2:**
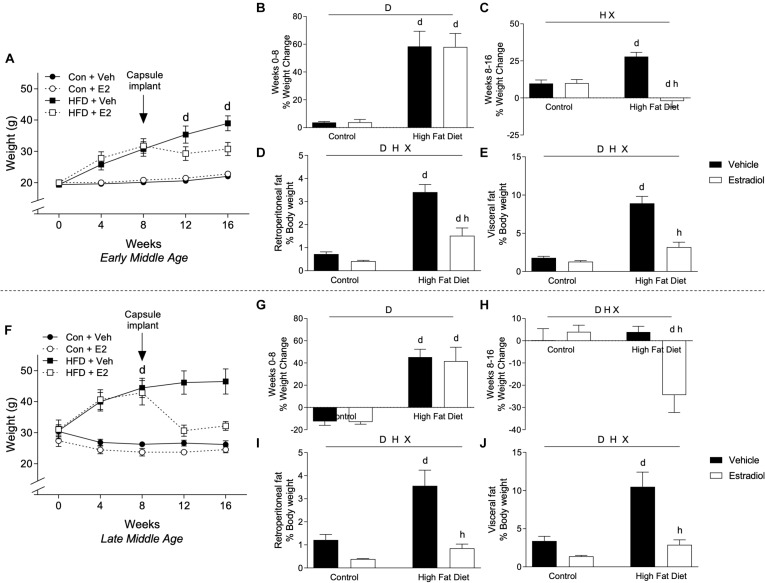
Body weight changes in early- and late-MA female 3xTg-AD mice. Body weight was measured weekly in **(A)** early-MA females and **(F)** late-MA females. Estradiol-filled capsules were administered after 8 weeks of diet. The percent weight change was calculated over the first 8 weeks of diet, before capsule implant, in **(B)** early-MA and **(G)** late-MA mice. Change in body weight was also measured during the second 8 weeks, to assess the effect of E2 treatment, in **(C)** early-MA and **(H)** late-MA female mice. Retroperitoneal and visceral fat pads were weighed and normalized to body weight in **(D,E)** early-MA and **(I,J)** late-MA mice, respectively. Data from early-MA (upper panels) and late-MA (lower panels) is separated by a dotted line. Significance is *p* < 0.05. *D* = significant main effect of diet; *H* = significant main effect of hormone treatment; *X* = significant interaction between diet and hormone; *d* = significant *post hoc* effect between diets, but within hormone treatment; *h* = significant *post hoc* effect between hormone treatment, within diet.

By week 8 of the experimental period, HFD was associated with significantly increased body weight relative to Con in both the early-MA and late-MA groups [Figure 2A, *F*_diet_(1,28) = 41.4, *p* < 0.0001; 2B, *F*_diet_(1,28) = 54.5, *p* < 0.0001; 2F, *F*_diet_(1,23) = 35.1, *p* < 0.0001; and 2G, *F*_diet_(1,23) = 52.5, *p* < 0.0001] (Table 1). In the early-MA groups, vehicle-treated mice on HFD continued to show significant weight gain throughout the next 8 weeks, whereas E2-treated mice exhibited no further increase in body weight although they remained significantly heavier than both groups of mice maintained on Con diet (Figure 2A). There was a significant main effect of hormone treatment and a significant interaction between diet and hormone treatment on percentage of weight change after the capsule implant [Figure 2C, *F*_hormone_(1,28) = 24.7, *p* < 0.0001; *F*_interaction_(1,28) = 25.7, *p* < 0.0001]. Interestingly, unlike the early-MA mice, the late-MA mice on HFD lost weight after implantation with E2 (Figure 2F). There were significant main effects of both diet and hormone treatment as well as an interaction between these two factors in the percent weight change in the late-MA animals [Figure 2H, *F*_diet_(1,23) = 5.5, *p* < 0.05; *F*_hormone_(1,23) = 5.4, *p* < 0.05; *F*_interaction_(1,23) = 9.3, *p* < 0.001]. There was no significant weight change difference after hormone treatment in animals of either reproductive age that received the Con diet (Table 1).

The effects of diet and hormone treatments on body weight were paralleled by differences across groups in the weights of fat depots. Within each age group, retroperitoneal (early-MA, Figure 2D and late-MA, Figure 2I) and visceral (early-MA, Figure 2E and late-MA, Figure 2J) fat pad weights (normalized to body weight) showed significant main effects of diet and hormone as well as an interaction between the two treatments (see Table 1 for statistical values). Specifically, HFD was associated with increased fat pad weight whereas E2 attenuated the HFD-induced increases [early-MA, Figure 2D, *F*_interaction_(1,28) = 10.7, *p* < 0.01; Figure 2E, *F*_interaction_(1,28) = 21.1, *p* < 0.0001; Figure 2I, *F*_interaction_(1,23) = 6.4, *p* < 0.05; and late-MA Figure 2J, *F*_interaction_(1,23) = 5.9, *p* < 0.05].

Data analyses were designed to determine the independent and interactive effects of HFD and E2 treatment in both early-MA and late-MA female mice. Although not a primary objective, the collected data can also be statistically analyzed to compare outcomes as a function of age (for complete statistical analysis, see Table 2). The initial body weights prior to experimental enrollment revealed that the late-MA animals started the experiment significantly heavier than the early-MA animals (Figure 2A vs. Figure 2F, *t*-test: *p* < 0.0001). Further, there was a significant main effect of age on weight gain in both the initial and final 8 weeks of the experiment [Figure 2B vs. Figure 2G, *F*_age × diet_(1,26) = 4.4, *p* < 0.05; and Figure 2C vs. Figure 2H, *F*_age × diet_(1,26) = 24.6, *p* < 0.0001]. The late-MA animals showed lower percent weight gain on both Con diet and HFD than the early-MA mice [Figures 2A vs. Figure 2F, *F*_time × age × diet_(4,104) = 55.8, *p* < 0.0001]. However, net increases in body mass (measured in grams) were similar, suggesting differences in percent weight gain largely reflect the differences in initial weights. Although both groups gained approximately the same total grams from 0 to 8 weeks, only the early-MA females on HFD continued to gain weight during weeks 8–16. Also, there was a significant effect of age on the response to E2, with late-MA animals losing significantly more weight than early-MA animals on HFD [Figure 2A vs. Figure 2F, *F*_time × age × hormone_(4,104) = 55.7, *p* < 0.0001]. There were no significant differences between the ages in the normalized weights of the fat pads (Figure 2D vs. Figure 2I and Figure 2E vs. Figure 2J).

### Estradiol Affects Metabolic Measures in Both Early and Late Middle Age

**FIGURE 3:**
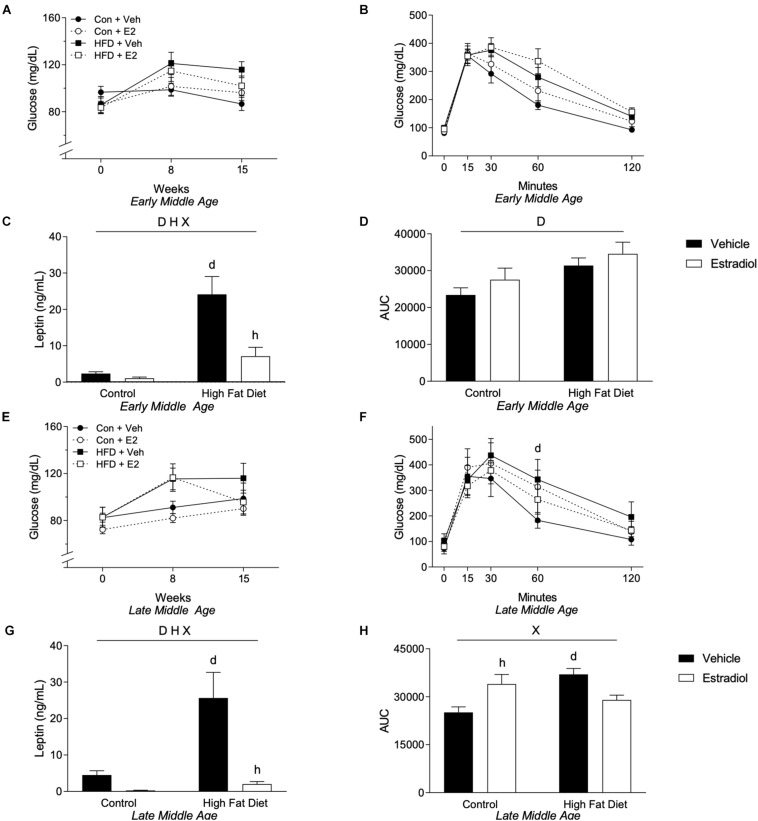
Metabolic effects in early- and late-MA female 3xTg-AD mice. Fasting glucose was measured at weeks 0, 8, and 15. Fasting glucose levels for **(A)** early-MA and **(E)** late-MA mice. An oral glucose tolerance test was administered at week 15 in **(B)** early-MA and **(F)** late-MA females. Fasting leptin was measured from the plasma collected at week 16 in **(C)** early-MA and **(G)** late-MA mice. The area under the curve (AUC) was calculated from the glucose tolerance test in **(D)** early-MA and **(H)** late-MA mice. Significance is *p* < 0.05. *D* = significant main effect of diet; *H* = significant main effect of hormone treatment; *X* = significant interaction between diet and hormone; *d* = significant *post hoc* effect between diets, but within hormone treatment; *h* = significant *post hoc* effect between hormone treatment, within diet.

Plasma levels of leptin determined at week 16 closely followed changes in adiposity. In both age groups, fasting leptin concentrations were significantly higher in HFD groups with ∼8-fold increase in the early-MA group and ∼5-fold elevation in the late-MA group [Figure 3C, *F*_diet_(1,28) = 25.3, *p* < 0.0001; and 3G, *F*_diet_(1,23) = 9.5, *p* < 0.01]. Further, E2 treatment robustly inhibited the HFD-associated increase in leptin levels. In early-MA, E2 was associated with ∼70% decrease in leptin relative to the vehicle-treated HFD group, whereas in late-MA the E2-induced decrease in leptin was ∼85% [Figure 3C, *F*_interaction_(1,28) = 8.1, *p* < 0.01; and Figure 3G, *F*_interaction_(1,23) = 6.8, *p* < 0.05] (Table 1).

To determine whether the observed HFD-induced increases in body weight, fat mass, and leptin levels were associated with functional metabolic impairments, we assessed both fasting glucose levels and glucose tolerance. In general, HFD diet was linked with increases in fasting glucose levels, which were measured at weeks 0, 8, and 15. There was a non-significant trend [Figure 3A, *F*_diet_(1,28) = 3.74, *p* = 0.06] of a main effect of diet and a significant interaction between time and diet on fasting glucose in the early-MA mice [Figure 3A, *F*_time × diet_(2,56) = 5.11, *p* < 0.01]. In the late-MA mice, there was a significant main effect of diet on fasting glucose [Figure 3E, *F*_diet_(1,23) = 6.5, *p* < 0.05]. Similarly, HFD was associated with impairments in glucose tolerance. There was a significant main effect of diet on glucose tolerance in the early-MA animals [Figure 3B, *F*_diet_(1,28) = 6.53, *p* < 0.05] that was also reflected in the area under the curve measurements [Figure 3D, *F*_diet_(1,28) = 8.0, *p* < 0.01]. In the late-MA mice, HFD resulted in poorer glucose tolerance that was significantly different from Con at the 60 min time point [Figure 3F, *F*_time × diet × hormone_(4,80) = 3.15, *p* < 0.05]. Further, there were significant interactive effects between diet and hormone treatment on the area under the curve [Figure 3H, *F*_interaction_(1,20) = 16.1, *p* < 0.001] such that HFD significantly impaired glucose tolerance specifically in vehicle-treated mice (*p* < 0.05) and E2 worsened glucose tolerance in the Con diet (*p* < 0.05).

We also considered the independent effects of age on metabolic outcomes. In general, metabolic consequences of HFD and E2 treatments were similar with HFD generally leading to a poorer metabolic profile regardless of chronological age and E2 improving some of the metabolic changes induced by HFD, including fasting glucose. However, there was a non-significant trend for an age × diet interaction in the GTT AUC with late-MA animals in all groups appearing to have a similar or greater AUC compared to the early-MA animals [Figure 3D vs. Figure 3H, *F*_age × diet_(1,24) = 3.5, *p* = 0.08].

### Behavioral Performance Is Improved by Estradiol Treatment Only in Early-MA Mice

**FIGURE 4:**
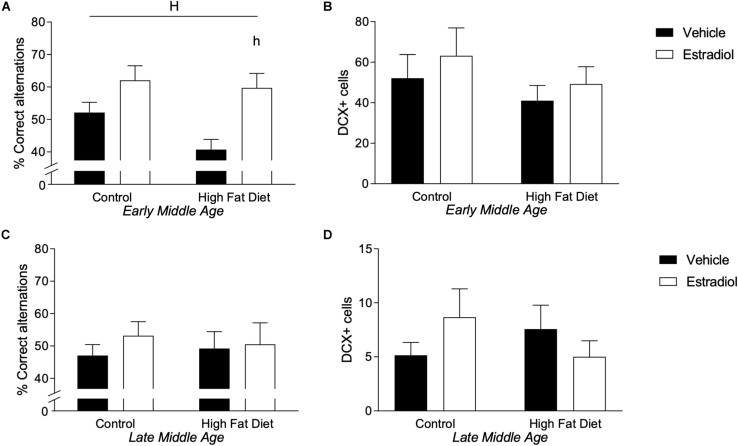
Behavior and neurogenesis in early- and late-MA female 3xTg-AD mice. Correct responses in the spontaneous alternation test were measured in **(A)** early-MA and **(C)** late-MA 3xTg-AD females at week 14. Doublecortin-positive cells were counted in the dentate gyrus of the hippocampus at week 16 in **(B)** early-MA and **(D)** late-MA mice to measure neurogenesis. Significance is *p* < 0.05. *H* = significant main effect of hormone treatment; *h* = significant *post hoc* effect between hormone treatment, within diet.

HFD in mice has been associated with impairments (Winocur and Greenwood, 2005; Park et al., 2010; McLean et al., 2018) and E2 with improvements (Carroll et al., 2007; Barker and Galea, 2008; Christensen and Pike, 2017) in both hippocampal-dependent cognitive tasks and hippocampal neurogenesis. To assess these outcomes, hippocampal-dependent cognitive function was assessed by spontaneous alternation behavior (SAB) and neurogenesis by levels of doublecortin (DCX)-immunoreactive cells in the dentate gyrus. In early-MA females, there was a statistically non-significant trend toward reduced SAB performance by HFD and a significant main effect of hormone treatment such that E2 was associated with behavioral improvement [Figure 4A, *F*_hormone_(1,22) = 12.9, *p* < 0.01]. Indeed, early-MA mice maintained on HFD and treated with E2 showed the greatest improvement with significantly better SAB performance than the HFD vehicle-treated animals (*p* < 0.05). These effects appear to be unrelated to neurogenesis as there were no significant differences in numbers of DCX-immunoreactive cells across early-MA groups (Figure 4B). In late-MA mice, there were neither significant main effects nor significant interactions of diet and hormone treatments on SAB and DCX-immunoreactive cell number (Figures 4C,D).

There was no significant effect of age on SAB, although a greater percentage of the late-MA mice (22.7%) scored less than 40% correct alternations, which is uncommon in the early-MA animals (12.5%), where it occurred almost exclusively in the HFD group. The total number of DCX-positive cells was significantly reduced ∼5–10-fold in the late-MA animals compared to the early-MA mice [Figure 4B vs. Figure 4D, *F*_age × diet_(1,26) = 28.5, *p* < 0.0001] (see Table 2).

### Effects of Diet and Estradiol Treatment on Alzheimer-Related Neuropathology

**FIGURE 5:**
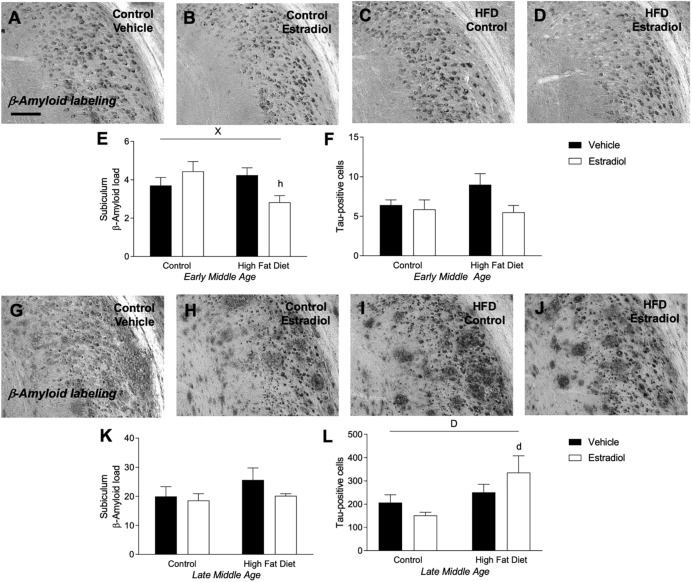
Measures of Alzheimer-related neuropathology in early- and late-MA female 3xTg-AD mice. Representative pictures of β-amyloid immunolabeling in the subiculum of the hippocampal formation from each treatment group in **(A–D)** early-MA and **(G–J)** late-MA female mice. β-Amyloid load was measured in the subiculum of **(E)** early-MA and **(K)** late-MA. Tau-immunolabeled cells were counted in the subiculum and CA1 hippocampal regions in **(F)** early-MA and **(L)** late-MA mice. Significance is *p* < 0.05. *D* = significant main effect of diet; *H* = significant main effect of hormone treatment; *X* = significant interaction between diet and hormone; *d* = significant *post hoc* effect between diets, but within hormone treatment; *h* = significant *post hoc* effect between hormone treatment, within diet.

The two primary neuropathological hallmarks of AD, deposition of Aβ and abundant neurons with hyperphosphorylated tau (Iqbal et al., 2009; Crews and Masliah, 2010; Vinters, 2015), are increased with age in 3xTg-AD mice (Oddo et al., 2003). Aβ deposition was measured by quantifying Aβ immunoreactive load, and tau pathology was measured by counts of cells immunolabeled with AT8 antibody that specifically recognizes hyperphosphorylated tau species. In the 3xTg-AD mouse model of AD, accumulations of tau and Aβ in the subiculum increased significantly with age, as reported previously (Oddo et al., 2003). Tau increased ∼20-fold in the late-MA animals compared to early-MA females [Figure 5L vs. Figure 5F, *F*_age × diet_(1,26) = 57.2, *p* < 0.0001]. Aβ load was increased ∼5-fold in the late-MA animals [Figure 5K vs. Figure 5E, *F*_age × diet_(1,26) = 94.4, *p* < 0.0001].

In early-MA mice, there was a significant interaction between diet and hormone treatment on Aβ load [Figures 5A–E, *F*_interaction_(1,28) = 9.9, *p* < 0.01] in which E2 treatment was associated with reduced Aβ burden in the subiculum specifically in the context of HFD (*p* < 0.05). There were no significant effects of diet or hormone on Aβ load in hippocampus CA1 (data not shown). Numbers of AT8-immunoreactive cells in subiculum showed a statistically non-significant trend of a main effect of hormone treatment (*p* = 0.07) with E2 decreasing tau pathology that was most apparent in the presence of HFD [Figure 5F, *F*_hormone_(1,27) = 3.4, *p* = 0.07]. In the late-MA animals, there were no significant main effects of diet or hormone treatment on Aβ load in either subiculum (Figures 5G–J,K) or hippocampus CA1 (data not shown). Numbers of AT8-immunoreactive cells showed a significant main effect of diet in which HFD was associated with more hyperphosphorylated tau-positive cells, particularly in the presence of E2 treatment [Figure 5L, *F*_diet_(1,23) = 6.3, *p* < 0.05].

### Estradiol Reduces Microglial Activation in Early-MA but Not Late-MA Mice

**FIGURE 6:**
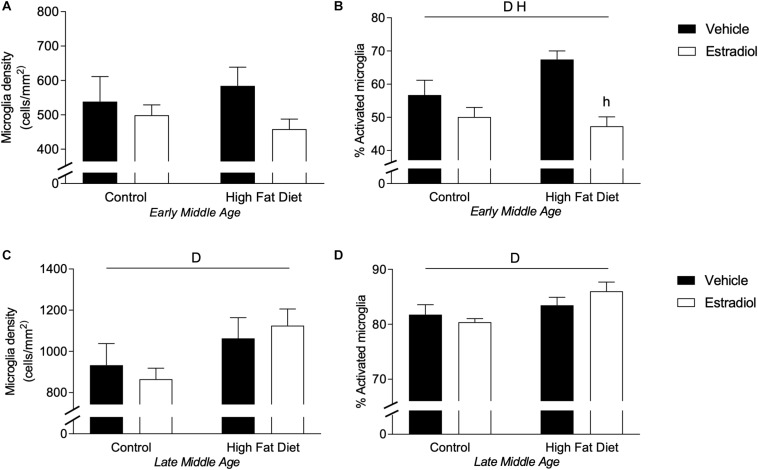
Microglial density and activation in the hippocampus of female 3xTg-AD mice. Microglia in the hippocampus were immunostained with Iba-1 antibody, counted, and morphologically characterized as either activated or resting. Microglia cell density was measured in **(A)** early-MA and **(C)** late-MA mice. The percent of microglia exhibiting activated morphology was determined in **(B)** early-MA and **(D)** late-MA mice. Significance is *p* < 0.05. *D* = significant main effect of diet; *H* = significant main effect of hormone treatment.

Microglial activation is modulated by E2 (Yun et al., 2018; Yilmaz et al., 2019) and is implicated in both regulation of AD-related pathology (Webers et al., 2019) and mediating adverse neural effects of HFD (Thaler et al., 2012; Kim et al., 2019). To investigate potential microglial contributions to observed effects, we quantified microglia cell number and activation phenotype in hippocampus as has been described previously (Christensen and Pike, 2017; Moser and Pike, 2017). In early-MA mice, microglia number showed no significant differences across groups (Figure 6A). However, there were significant main effects of both diet and hormone treatment on microglial activation state such that HFD was associated with increased activation, which was significantly reduced by E2 treatment [Figure 6B, *F*_interaction_(1,28) = 4.2, *p* < 0.05; HFD + Veh vs. HFD + E2, *p* < 0.001]. In late-MA animals, there were significant main effects of diet on both microglia number [Figure 6C, *F*_diet_(1,22) = 4.8, *p* < 0.05] and activation phenotype [Figure 6D, *F*_diet_(1,22) = 6.0, *p* < 0.05] with HFD-treated mice exhibiting greater microglial density and a higher proportion of activated microglia in the hippocampus than mice maintained on control diet. There were no significant effects of E2 treatment on microglial measures in late-MA mice (Table 1).

There was a significant effect of aging and an age X diet interaction on microglial number [Figure 6A vs. Figure 6C, *F*_age × diet_(1,25) = 28.0, *p* < 0.0001]. Indeed, aging nearly doubled the density of microglia in the hippocampus. The percentage of activated microglia was also significantly increased by aging with an age × diet and age × hormone interaction [Figure 6A vs. Figure 6C, *F*_age × diet_(1,25) = 44.6, *p* < 0.0001; *F*_age × hormone_(1,26) = 141.3, *p* < 0.0001] (see Table 2). The number and activation state of microglia are likely correlated with the increased Aβ load in late-MA animals, but it is unclear if microglia changes are a cause or effect of amyloid changes.

### HFD Increases Peripheral Tissue Inflammation in Early-MA and Late-MA Mice

**FIGURE 7:**
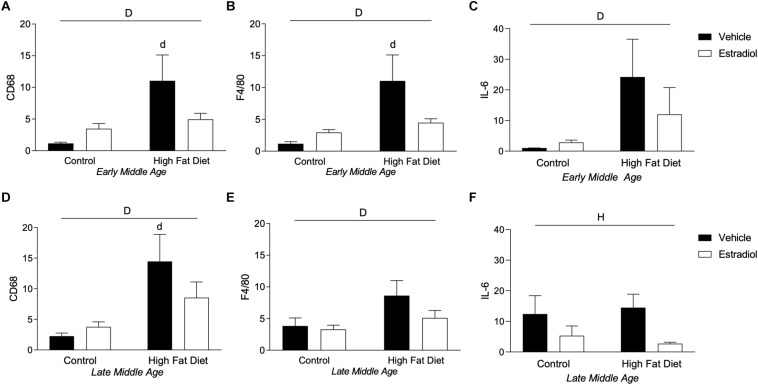
Macrophage markers in adipose tissue in early- and late-MA female 3xTg-AD mice. Relative mRNA expression of the macrophage markers CD68 and F4/80 was measured in visceral adipose tissue from **(A,B)** early-MA and **(D,E)** late-MA mice, respectively. The cytokine IL-6 mRNA expression was measured from adipose tissue in **(C)** early-MA and **(F)** late-MA mice. Significance is *p* < 0.05. *D* = significant main effect of diet; *H* = significant main effect of hormone treatment; *d* = significant *post hoc* effect between diets, but within hormone treatment.

Diet-induced obesity results in macrophage infiltration and activation in peripheral tissues including adipose tissue that yields an inflammatory state (Huh et al., 2014), an effect that can be attenuated by E2 (Stubbins et al., 2012; Davis et al., 2013). To assess obesity-related macrophage infiltration in visceral adipose tissue, mRNA expression of the macrophage markers CD68 and F4/80 was quantified. In both the early-MA and late-MA groups, HFD significantly increased expression of both macrophage markers [Figure 7A, *F*_diet_(1,28) = 7.1, *p* < 0.05; Figure 7B, *F*_diet_(1,28) = 7.5, *p* < 0.05; Figure 7D, *F*_diet_(1,23) = 9.8, *p* < 0.01; and Figure 7E, *F*_diet_(1,23) = 4.4, *p* < 0.05]. There were no statistically significant effects of E2 treatment on the HFD-associated increases in CD68 and F4/80 in early-MA and late-MA mice. In addition to macrophage markers, the levels of the pro-inflammatory cytokine IL-6 were assessed by PCR. In early-MA animals, IL-6 expression was significantly increased by diet [Figure 7C, *F*_diet_(1,28) = 4.6, *p* < 0.05] and in late-MA animals IL-6 was significantly reduced by estradiol treatment but was not significantly affected by diet [Figure 7F, *F*_hormone_(1,23) = 5.2, *p* < 0.05].

There was no significant, independent effect of aging on macrophage markers in visceral adipose tissue. However, when all the animals were compared together, a significant effect of diet and a significant interaction between diet and hormones was seen that was not apparent when the ages were compared separately [Figure 7A vs. Figure 7D, *F*_diet_(1,51) = 17.3, *p* < 0.001; *F*_diet × hormone_(1,51) = 5.4, *p* < 0.05; Figure 7B vs. Figure 7E, *F*_diet_(1,51) = 11.1, *p* < 0.01; *F*_diet × hormone_(1,51) = 4.4, *p* < 0.05]. When comparing adipose levels of IL-6 across aging, there is a trend for HFD to increase IL-6 levels and a statistically non-significant trend toward an age X diet interaction in which early-MA animals show a greater response to diet than late-MA animals [Figure 7C vs. Figure 7F, *F*_diet_(1,51) = 3.1, *p* = 0.09; *F*_age × diet_(1,51) = 3.3, *p* = 0.08].

## Discussion

Obesity is a significant and highly prevalent risk factor for poor health outcomes and increased vulnerability to several diseases, including AD (Moser and Pike, 2016; Pugazhenthi et al., 2017; Alford et al., 2018). Like other AD risk factors, the deleterious effects associated with obesity can be positively and negatively modulated by several factors, underscoring the need to understand how obesity interacts with other regulators of AD. In this study, we looked at how obesity, aging, and estradiol interact to affect AD neuropathology. In particular, this study assessed the protective effects of E2 against metabolic and AD-related outcomes associated with obesity during the initial versus late stages of middle age in female 3xTg-AD mice. Prior related studies largely delivered E2 simultaneously with HFD (Litwak et al., 2014; Yasrebi et al., 2017), and thereby assessed the preventive effects of E2 against obesity. Here, E2 was initiated after the establishment of diet-induced obesity, which considers potential treatment effects of E2 in the context of an existing AD risk factor. In early-MA, we observed that E2 effectively reduced systemic effects of obesity, improved working memory performance, lessened Aβ burden in subiculum, and decreased an activated microglial phenotype. In late-MA, metabolic benefits of E2 were also found, however there was an absence of protection against AD-related neuropathology and cognitive impairment.

Metabolic benefits of estrogens may be retained during aging. The depletion of estrogens during menopause is associated with numerous changes throughout the body, including increased adiposity and associated metabolic impairments (Wend et al., 2012). Reduced estradiol levels during the menopause transition have been linked to increased risk of type 2 diabetes (Park et al., 2017). Postmenopausal women treated with HT exhibit reduced adiposity (Papadakis et al., 2018) and metabolic benefits including decreases in fasting glucose and plasma lipids and reduced type 2 diabetes risk (Andersson et al., 1997; Kanaya et al., 2003; Margolis et al., 2004; Mauvais-Jarvis et al., 2017). Our data show that late-MA mice had significantly higher mass than early-MA animals at the start of the experiment, suggesting that the combination of reproductive and chronological aging in female mice models aspects of the increased adiposity observed in postmenopausal women. More importantly, E2 administered after the development of obesity was able to mitigate weight gain both in early-MA and late-MA animals, suggesting retained functionality of peripheral estrogen signaling across middle age. Interestingly, late-MA animals showed a greater response to E2 than early-MA mice in terms of body mass. In addition, plasma leptin concentration was significantly increased by diet and reduced by E2 in both early-MA and late-MA mice. These data complement prior findings that E2 treatment initiated simultaneously with obesogenic diet attenuated adiposity in advanced middle-aged female mice and that E2 late in life can reduce metabolic dysfunction induced by HFD (Bryzgalova et al., 2008; Christensen and Pike, 2017). However, recent studies in aged mice (Farhadi et al., 2020) and macaques (Purnell et al., 2019) maintained on an obesogenic diet indicate loss of E2 efficacy in reducing body mass and improving metabolic measures. Unclear from both the human and animal literatures is the relationship between chronological and reproductive aging in regulating E2 efficacy and how this may vary across tissues. Prior work has demonstrated the importance of reproductive over chronological aging for some neural measures in early middle-aged female rats (Yin et al., 2015) and women (Mosconi et al., 2017). Our current observations that late-MA mice retain beneficial E2 effects for peripheral metabolic outcomes but not neural endpoints suggest key differences across tissues. These are critical issues that require additional study.

The neuroprotective actions of estrogens appear to be adversely affected by chronological and reproductive aging. In women, there is an extensive but conflicting literature concerning the ability of HT to reduce risks of AD and related dementias. Although the controversy has yet to be resolved definitively, a leading perspective is that neuroprotective estrogen actions are diminished across the menopause transition such that there may be a several year window of opportunity, beginning at perimenopause, during which HT exhibits transient neural efficacy (Maki, 2006; Henderson et al., 2016). After this period, HT is thought to exert neutral or perhaps harmful neural effects (Henderson et al., 2005; Whitmer et al., 2011; Shao et al., 2012). Experimental animal studies parallel aspects of the human literature in that E2 treatment yields significant neural benefits in middle-aged females across several rodent paradigms (Pike et al., 2009), outcomes that are often attenuated in aged animals (Aenlle and Foster, 2010; Liu et al., 2015). Note, however, that E2 can exert positive neural effects, including improved behavioral performance, even in aged female mice (Frick et al., 2002; Vaucher et al., 2002). Unclear is whether and how obesity affects the trend of age-related decreases in neural efficacy of E2. In the current study, E2 treatment in early-MA mice under Con diet yielded only statistically non-significant trends toward improved behavioral performance and reduced Aβ. These observations are in contrast to our previous findings of E2 in gonadally intact (Christensen and Pike, 2017) and ovariectomized (Carroll et al., 2007) female 3xTg-AD mice, perhaps owing to the comparatively short treatment period in the present study. In the context of HFD, early-MA mice treated with E2 showed improved behavior, reduced Aβ burden in subiculum, and a non-significant trend toward lower phospho-tau labeling. Importantly, late-MA mice treated with E2 showed a quite different pattern with no improvements in behavior and Aβ and a significant worsening of phospho-tau. Together, these findings suggest that neuroprotective effects of E2 are retained in early-MA even in the presence of a preexisting obese state. In apparent contrast to our observations, recent work in aged female macaques showed that positive effects of E2 on object recognition memory were blunted by obesogenic diet (Bethea et al., 2017). This lack of concordance suggests the possibility of a complex relationship between obesity and E2 neural efficacy during aging that may be affected by several parameters, including specific aspects of diet, aging, and neural measure.

Coincident impairments in metabolism and brain functions suggest possible mechanistic relationships. Indeed, metabolic dysfunction has been widely hypothesized to be a key mechanism by which obesity induces neural deficits and increases AD risk (O’Brien et al., 2017; Pugazhenthi et al., 2017; Alford et al., 2018). Consistent with this position, previous work has shown interventions that reduce obesity in transgenic mouse models of AD also improve behavioral and pathological outcomes (Maesako et al., 2012; Walker et al., 2017; Yeh et al., 2017; Ettcheto et al., 2018). However, the current findings are not supportive of direct associations between improved metabolic profile and reduced AD-related indices. E2 significantly reduced body mass, adiposity, and circulating leptin levels in both early-MA and late-MA yet was associated with improved behavioral performance and lower Aβ burden only in early-MA. The observation that metabolic but not neural benefits of E2 persisted in late-MA argues against metabolic regulation as a primary component underlying E2 protection against AD-related outcomes.

Aside from improving metabolic indices, estrogens may also combat neural consequences of obesity by regulating systemic and CNS inflammatory tone. Diet-induced obesity results in activated microglial phenotypes, which in turn mediate, at least in part, damaging, pro-inflammatory effects of obesity on the brain (Hao et al., 2016; André et al., 2017). Importantly, impaired microglial functions are increasingly implicated as key regulators of AD pathogenesis (Kaur et al., 2019; Webers et al., 2019). Estradiol represents a compelling approach in combating effects of obesity because it can attenuate disease-promoting microglial actions both directly by regulating microglia (Villa et al., 2016; Butler et al., 2020) and indirectly by reducing adiposity (Brown et al., 2010). We observed an obesity-induced increase in an activated microglial phenotype at both early-MA and late-MA. Importantly, E2 reversed this increase in microglial activation in early-MA but had no significant effect in late-MA. These data suggest that neural E2 regulation of microglia in female mice is sensitive to reproductive and/or chronological aging with efficacy largely absent by late middle-age. Interestingly, this pattern corresponds with the observed age-dependence of E2 protection against behavioral impairment and Aβ burden, suggesting perhaps that reduction of microglial activation may contribute to the mechanism(s) by which E2 protects against AD-related outcomes in obese female 3xTg-AD mice. Although estrogens are known to regulate peripheral inflammatory tone (Kovats, 2015; Klein and Flanagan, 2016), our limited assessment showed that E2 treatment of HFD mice yielded only a non-significant trend toward blunting the obesity-induced increase in macrophage markers in adipose in early-MA, which was less apparent in late-MA. These observations are not supportive of a possible mechanistic role of reduced peripheral inflammatory tone mediating neural effects of E2, though the restricted assessment of systemic inflammation status precludes a definitive conclusion.

In summary, these results add to an important and growing literature defining the impact of aging on the ability of estrogen-based therapies to protect against AD. The key finding is that, in the context of preexisting obesity, E2 treatment improves cognition and reduces Aβ burden in mice at chronological ages associated with early but not late reproductive senescence. This finding supports the “window of opportunity” position that HT in aging women is most likely to yield protective brain effects when initiated near perimenopause but absent or even deleterious outcomes when delivered several years after the menopause transition (Whitmer et al., 2011; Shao et al., 2012; Henderson et al., 2016). Further, because increasing adiposity is associated with chronological and reproductive aging in women, the findings provide preclinical evidence that HT may provide effective intervention for women that are overweight or obese, which are established AD risk factors. The results also provide clues to possible mechanisms contributing to E2 protection against obesity. To the extent that deleterious effects of obesity drive neural dysfunction as a result of systemic metabolic impairment, the current findings suggest that E2 may benefit brain health and reduce AD risk well into middle age as a consequence of its ability to lower adiposity and improve metabolic function in the context of obesity. However, the discordance between E2 protection against metabolic versus neural indices in late reproductive senescence argue that other actions, perhaps including regulation of microglial activation states, play more significant roles in protections against AD. Continued investigation of the interactions among E2, aging, obesity, and other AD risk factors promises to improve the selective use of HT to reduce dementia risk.

## Data Availability Statement

The datasets generated for this study are available on request to the corresponding author.

## Ethics Statement

The animal study was reviewed and approved by the Institutional Animal Care and Use Committee of the University of Southern California. Procedures were performed under the supervision of university veterinarians.

## Author Contributions

AC and CP designed the study, analyzed the results, and wrote the manuscript. AC performed the experiments. JL conducted and analyzed some of the PCR experiments.

## Conflict of Interest

The authors declare that the research was conducted in the absence of any commercial or financial relationships that could be construed as a potential conflict of interest.
